# Mendelian randomization unraveled: gender-specific insights into obesity-related phenotypes and colorectal cancer susceptibility

**DOI:** 10.3389/fendo.2024.1322253

**Published:** 2024-06-06

**Authors:** Xinyi Chen, Mu Yang, Weiheng Zhao, Jingyao Tu, Qingxu Liu, Xianglin Yuan

**Affiliations:** Department of Oncology, Tongji Hospital, Tongji Medical College, Huazhong University of Science and Technology, Wuhan, Hubei, China

**Keywords:** obesity-related phenotypes, Mendelian randomization, colorectal cancer, waist-hip ratio, WHRadjBMI, causal relationship

## Abstract

**Objective:**

Evidence has been increasingly pointing towards a potential link between phenotypes related to obesity and the incidence of colorectal cancer. However, confirming this as a direct causal connection remains elusive. This investigation aims to elucidate the causative links between obesity-associated phenotypes and the incidence of colorectal cancer.

**Methods:**

Employing the Two Sample Mendelian Randomization (TwoSampleMR) R package, analyses were conducted using Mendelian randomization (MR) to discern potential causative links between obesity categories sourced from both the Institute for Education and University (IEU) Open GWAS Project and Zenodo, and colorectal tumors (data obtained from IEU Open GWAS and FinnGen). For primary evaluations, the study utilized the Wald ratio and the Inverse Variance Weighting (IVW) methods, while the MR-Egger approach was integrated for sensitivity assessment. Bidirectional Mendelian Randomization (Bidirectional MR), as well as Linkage Disequilibrium (LD) Score Regression with well-imputed HapMap3 single nucleotide polymorphisms (SNPs), were additionally executed. Sensitivity assessments entailed IVW, MR-Egger methodologies to assess heterogeneity and pleiotropy, along with a leave-one-out strategy. Instrumental variables were chosen judiciously based on predetermined P-value thresholds and F-statistics.

**Results:**

Results from MR evaluations did not identify a clear causative link between BMI and colorectal malignancy. Conversely, both measures of obesity, the Waist-Hip Ratio (WHR) and its adjusted form for BMI (WHRadjBMI), displayed a connection to increased risk of colorectal cancer, especially prominent among female subjects. Reverse MR analyses dismissed potential reverse causality between colorectal malignancies and obesity. A significant genetic interplay was observed between WHR, WHRadjBMI, and colorectal cancer instances. Ensuing MR probes spotlighted inflammatory bowel ailment as a protective factor, while salad intake was indicated as a potential risk concerning colorectal malignancies. Sensitivity reviews, which included tests for both pleiotropy and heterogeneity, validated the robustness of the MR findings.

**Conclusion:**

Findings from this research indicate that specific obesity-related parameters, notably WHR and WHRadjBMI, carry a causal relationship with an elevated colorectal cancer risk. The impact is distinctly more evident among females. Such insights might be pivotal for public health deliberations, hinting that individuals boasting a high WHR might necessitate intensified colorectal cancer screenings.

## Introduction

Worldwide, colorectal tumors (CRC) stand out as ranking third in cancer prevalence and occupying the position of the second most frequent driver behind cancer-related deaths (1). Even with advancements in both diagnosis and treatment methodologies, a significant surge in CRC occurrences was observed (2, 3), reinforcing the urgency of potent preventive approaches. Simultaneously, the worldwide increase in obesity, exemplified by a rising Body Mass Index (BMI) among adults, became a pivotal research focus (4). Statistics from the World Health Organization (WHO) highlighted an alarming trend, with approximately 1.5 billion adults worldwide classified as overweight and a significant 500 million deemed obese (5). Notably, a concurrent trend associated rising obesity levels with a heightened incidence of CRC (2, 6).

In obesity evaluation, both BMI and Waist-Hip Ratio (WHR) were highlighted as crucial modifiable determinants associated with CRC (7–9). Notably, obesity accounted for an estimated 4.6% of cancer-related mortalities worldwide (10). Furthermore, weight loss appeared to confer some protective benefits, especially among postmenopausal women (11). Associations between obesity and CRC emerged as intricate, shaped by several intertwined determinants. These encompassed elevations in insulin and IGF-1 pathways, growth factors, persistent inflammation, disruptions in hormonal equilibrium, adipokines, and fluctuations in sex hormone concentrations (12–14). Although these connections were reported, variations existed across different scientific publications. Distinct studies emphasized an elevated mortality risk among CRC patients possessing elevated BMI relative to those with a standard BMI (15, 16). Particularly, studies presented by Baade et al. (17) in conjunction with Kuiper et al. (18) demonstrated an augmented rate of deaths related to CRC in individuals with excessive weight, with recorded increases of 25% and 55% respectively. Yet, contrasting studies suggested that elevated BMI was not linked with increased mortality and could even be associated with reduced mortality risks in CRC patients (19, 20). These inconsistent results illuminated the intricacies and challenges in understanding the obesity-CRC relationship. Epidemiological data were often compromised by confounding variables, and their interpretive scope was additionally narrowed by issues of reverse causality (21). Furthermore, the study of other obesity-related metrics, like WHR, within the CRC context remained insufficient (22). These limitations underscored the pressing need for comprehensive, well-designed prospective studies to unravel the complex relationship between obesity and CRC.

In epidemiological research, Mendelian Randomization (MR) is esteemed as an invaluable tool, leveraging genetic markers to determine causative relationships between risk determinants and health outcomes (23). Within this framework, causal inferences are further refined by phenotype-specific genetic variants (24). Crucially, MR overcomes challenges frequently associated with observational analysis, including confounding and issues tied to causality reversal, thus facilitating the assessment of enduring risk factors through a wide range of health outcomes (25, 26).

For the study at hand, exhaustive evaluations were undertaken using two-sample MR and Genetic Correlation methodologies to determine the causative ties between obesity indicators—specifically BMI and WHR—and the susceptibility to CRC. Aggregate data was sourced from comprehensive Genome-Wide Association Studies (GWAS) focusing on obesity and CRC. Particular attention was given to the implications of BMI and WHR adjusted for BMI (WHRadjBMI) in cancer vulnerability. This consideration stemmed from previous claims positing that genetic markers associated with WHR might delineate more obscure biological pathways than those linked to BMI or WHRadjBMI (27). Consistent with prevailing research conclusions, it was hypothesized that obesity metrics, particularly BMI and WHR, had a causative relationship with CRC risk. Employing MR as an analytical approach in this study provided a method less prone to the biases observed in traditional observational research. The research focused on deriving insights vital for the development of future health prevention approaches and the definition of public health guidelines.

## Materials and methods

### Obesity-related GWAS information acquisition

Data, sorted based on obesity classification, was sourced from the IEU Open GWAS Project platform at https://gwas.mrcieu.ac.uk/. Distinct codes for these datasets include: ieu-a-90 for Obesity class 1, ieu-a-91 indicating Obesity class 2, and ieu-a-92 marking Obesity class 3. Within Obesity class 1, a count of 32,858 cases was observed, with the total sample reaching 98,697. For Obesity class 2, 9,889 cases were identified within a 72,546 sample. Obesity class 3 had 2,896 cases among a sample of 50,364 (28). Other datasets pertaining to obesity metrics were derived from https://zenodo.org/record/1251813. This array encompasses: Waist-Hip Ratio adjusted for Body Mass Index (WHRadjBMI), WHRadjBMI for females (WHRadjBMI_females), WHRadjBMI for males (WHRadjBMI_males); The raw Waist-Hip Ratio (WHR), WHR limited to females (WHR_females), and WHR restricted to males (WHR_males); Standard Body Mass Index (BMI) values, female-specific BMI (BMI_females), and male-specific BMI (BMI_males) (29). Details alongside the requisite download links have been itemized in **Table 1**.

**Table 1:** GWAS Data Summary for Obesity and Colorectal Cancer.

Identifier	Variable Description	Number of Cases	Sample Size	Download URL	References
ieu-a-90	Obesity class 1	32858	98697	https://gwas.mrcieu.ac.uk/	10.1038/ng.2606
ieu-a-91	Obesity class 2	9889	72546	https://gwas.mrcieu.ac.uk/	10.1038/ng.2606
ieu-a-92	Obesity class 3	2896	50364	https://gwas.mrcieu.ac.uk/	10.1038/ng.2606
whradjbmi	waist-to-hip ratio adjusted for body mass index	/	694649	https://zenodo.org/record/1251813	10.1093/hmg/ddy327
whradjbmi_females	waist-to-hip ratio adjusted for body mass index, Female samples only	/	379501	https://zenodo.org/record/1251813	10.1093/hmg/ddy327
whradjbmi_males	waist-to-hip ratio adjusted for body mass index, Male samples only	/	315284	https://zenodo.org/record/1251813	10.1093/hmg/ddy327
whr	waist-to-hip ratio	/	697734	https://zenodo.org/record/1251813	10.1093/hmg/ddy327
whr_females	waist-to-hip ratio, Female samples only	/	381152	https://zenodo.org/record/1251813	10.1093/hmg/ddy327
whr_males	waist-to-hip ratio, Male samples only	/	316772	https://zenodo.org/record/1251813	10.1093/hmg/ddy327
bmi	body mass index	/	806834	https://zenodo.org/record/1251813	10.1093/hmg/ddy327
bmi_females	body mass index, Female samples only	/	434794	https://zenodo.org/record/1251813	10.1093/hmg/ddy327
bmi_males	body mass index, Male samples only	/	374756	https://zenodo.org/record/1251813	10.1093/hmg/ddy327
ieu-b-4965	Colorectal cancer	5657	377673	https://gwas.mrcieu.ac.uk/files/ieu-b-4965/ieu-b-4965.vcf.gz	/
finn-b-C3_COLORECTAL	Colorectal cancer	3022	218792	https://www.finngen.fi/en/access_results	/
ukb-d-C3_COLON	Malignant neoplasm of colon	2437	361194	https://gwas.mrcieu.ac.uk/files/ukb-d-C3_COLON/ukb-d-C3_COLON.vcf.gz	/
finn-b-C3_COLON	Malignant neoplasm of colon	1803	218792	https://www.finngen.fi/en/access_results	/

### Colorectal cancer outcome data

Information related to malignant colorectal tumors was sourced from two distinct platforms: the IEU Open GWAS Project and the FinnGen platform. Within these platforms, data were drawn from four specific cohorts. These are labeled as ukb-d-C3_COLON and ieu-b-4965 within the UK Biobank database, and finn-b-C3_COLON and finn-b-C3_COLORECTAL in the FinnGen database. It was noted that there was no observed sample overlap between the datasets from UK Biobank and the FinnGen database. A comprehensive description and relevant download links can be found in **Table 1**.

### Additional data

A literature review was conducted to identify risk factors related to colorectal cancer, excluding genetic factors, race, and gender. Identified were twenty probable risk determinants: habits like smoking; dietary choices such as consuming processed meat, red meat, and alcohol; a scarcity in consuming fruits and vegetables; the presence of obesity; levels of physical exertion; consuming whole grains, dietary fiber, dairy items, fish, and tree nuts; specific vitamins such as D and C; utilization of calcium enhancers, non-steroidal anti-inflammatory medications, or aspirin; undergoing hormone replacement during the menopausal phase; administering statins; existence of type 2 diabetes; and conditions like inflammatory bowel disease (30). Subsequently, GWAS data for thirteen of these risk factors were retrieved from the OpenGWAS database: smoking (ukb-b-223), processed meat (ukb-b-6324), frequency of alcohol consumption (ukb-b-5779), salad/raw vegetable intake (ukb-b-1996), fresh fruit intake (ukb-b-3881), whole grains (ukb-d-1448_3), dietary fiber (ukb-b-19085), vitamin C (ukb-b-19390), vitamin D (ukb-b-18593), calcium supplements (ukb-b-7043), hormone replacement therapy during menopause (ukb-b-18541), type 2 diabetes (ebi-a-GCST006867), and inflammatory bowel disease (ieu-a-294).

### Conducting the Mendelian randomization assessment

Analyses rooted in Mendelian randomization were carried out on datasets relating to obesity and colorectal cancer using the TwoSampleMR R package (Version 0.5.7, TwoSampleMR documentation) (31). During the Mendelian randomization, the choice of linear model computation hinged upon the count of instrumental variables retained for each obesity-linked trait. For cases utilizing a single tool variable, the method involving Wald ratios was deemed suitable. Conversely, when confronted with 2-3 such variables, preference was given to the model with Inverse Variance Weighted (IVW; employing fixed effects). In cases with greater than three instrumental factors, the IVW model incorporating multiplicative effects is preferred. Standard methods, such as MR Egger and Maximum Likelihood, alongside weight-focused evaluations, are factored into the assessment.

### Inverse Mendelian randomization analysis

An effort was undertaken to discern whether obesity serves as a cause or consequence of colorectal cancer, or if a genuine bidirectional causal relationship between the two variables exists. Genetic variations pertaining to both obesity and colorectal cancer were employed to rigorously assess the three putative causal scenarios: 1) obesity precipitates colorectal cancer; 2) colorectal cancer induces obesity; or 3) there exists a true bidirectional causative link connecting obesity and colorectal cancer. When evaluating outcomes from both perspectives, disparities in statistical power associated with instrumental variables (IVs) were meticulously factored in.

### Assessing genetic interplay in colorectal cancer instances

Utilizing Linkage Disequilibrium Score Regression (LDSC), a recognized instrument for ascertaining heritability and genetic correlation through genome-wide association scrutiny, the differentiation of genuine polygenic signals from potential confounders like population stratification and cryptic relatedness becomes feasible. Information on genome-wide association analysis linked to obesity has been acquired, along with data on colorectal cancer from distinctive sources, namely FinnGen and the UK Biobank. The genomic association of obesity with colorectal malignancies got determined using the LDSC software (Version 1.0.1, accessible via an online repository https://github.com/bulik/ldsc/wiki/LD-Score-Estimation-Tutorial) (32). Originating from the third cycle of the 1000 Genomes Project, pertinent LD scores related to European heritage have been gathered. In order to counteract any reduced statistical potency stemming from subpar imputation accuracy, the scrutiny was confined to well-imputed HapMap3 single nucleotide polymorphisms (SNPs). A significance determination for the correlation adopted a *P*-value cutoff of 0.05.

### Sensitivity analyses

Within this evaluation, heterogeneity and pleiotropy levels underwent meticulous scrutiny using MR. Employing both the IVW approach and the MR Egger method, heterogeneity was accurately gauged. In contrast, pleiotropy was exclusively assessed through the MR Egger method. Leave-one-out analyses were carried out, omitting individual SNPs to discern any key SNPs that might alter the general outcomes.

### Criteria for instrumental variable selection

In the initial phase of the present investigation, instrumental variables associated with the exposure variable were meticulously screened based on the subsequent criteria:

A P-value threshold of less than 5e-8 was imposed,
An F-statistic exceeding 10 was required,
Linkage disequilibrium was eradicated within a 10,000kb window and an r2 value of 0.001,
A minimum allele frequency exceeding 0.01 was mandated.

## Results

### Mendelian randomization outcomes concerning obesity and colorectal cancer

**Figure 1:**
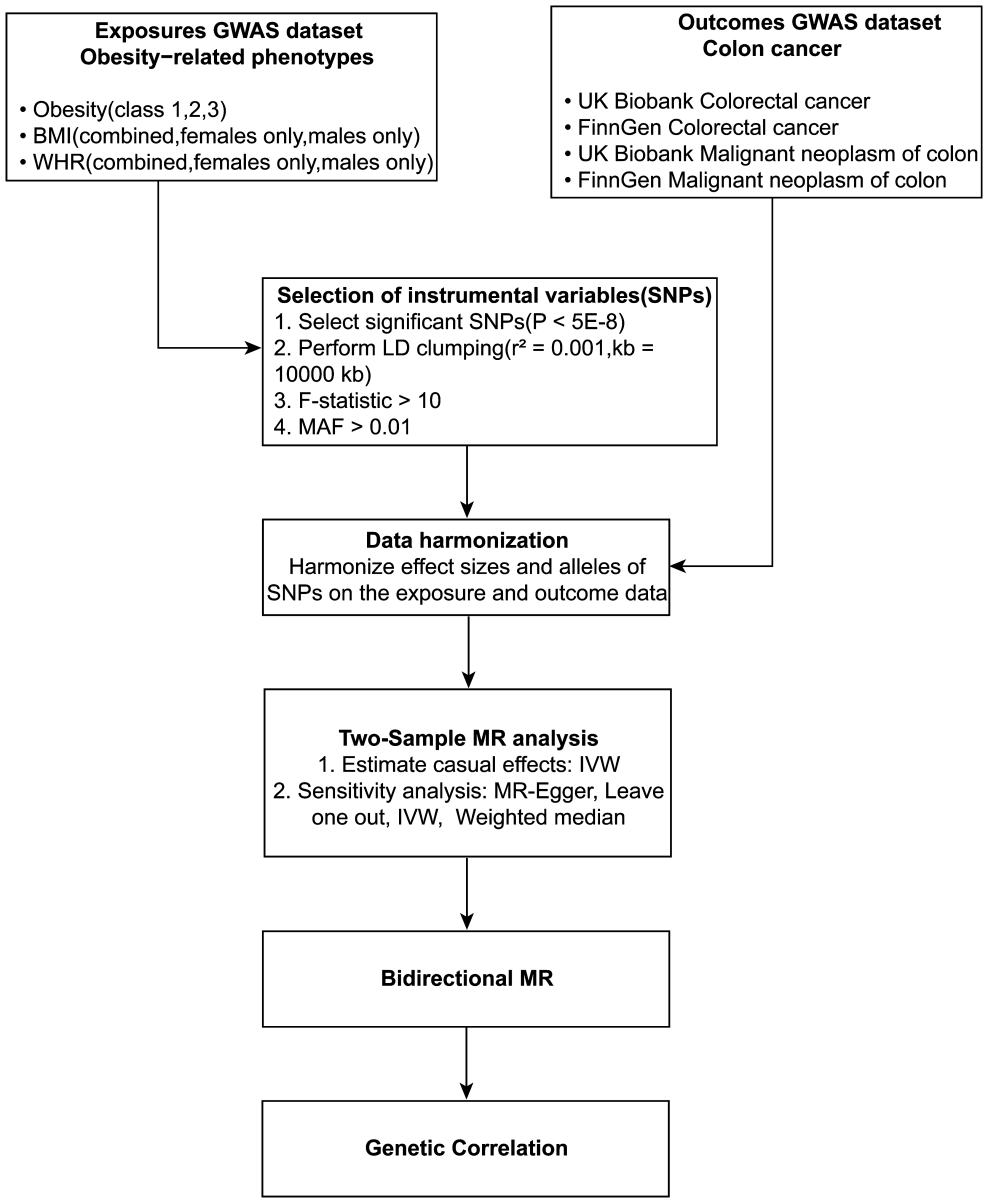
Schematic diagram of the study design. Depicted in the flowchart is the incorporation of obesity-linked SNPs as determinant variables and colorectal cancer sets as consequential variables in the MR analysis.

**Figure 2:**
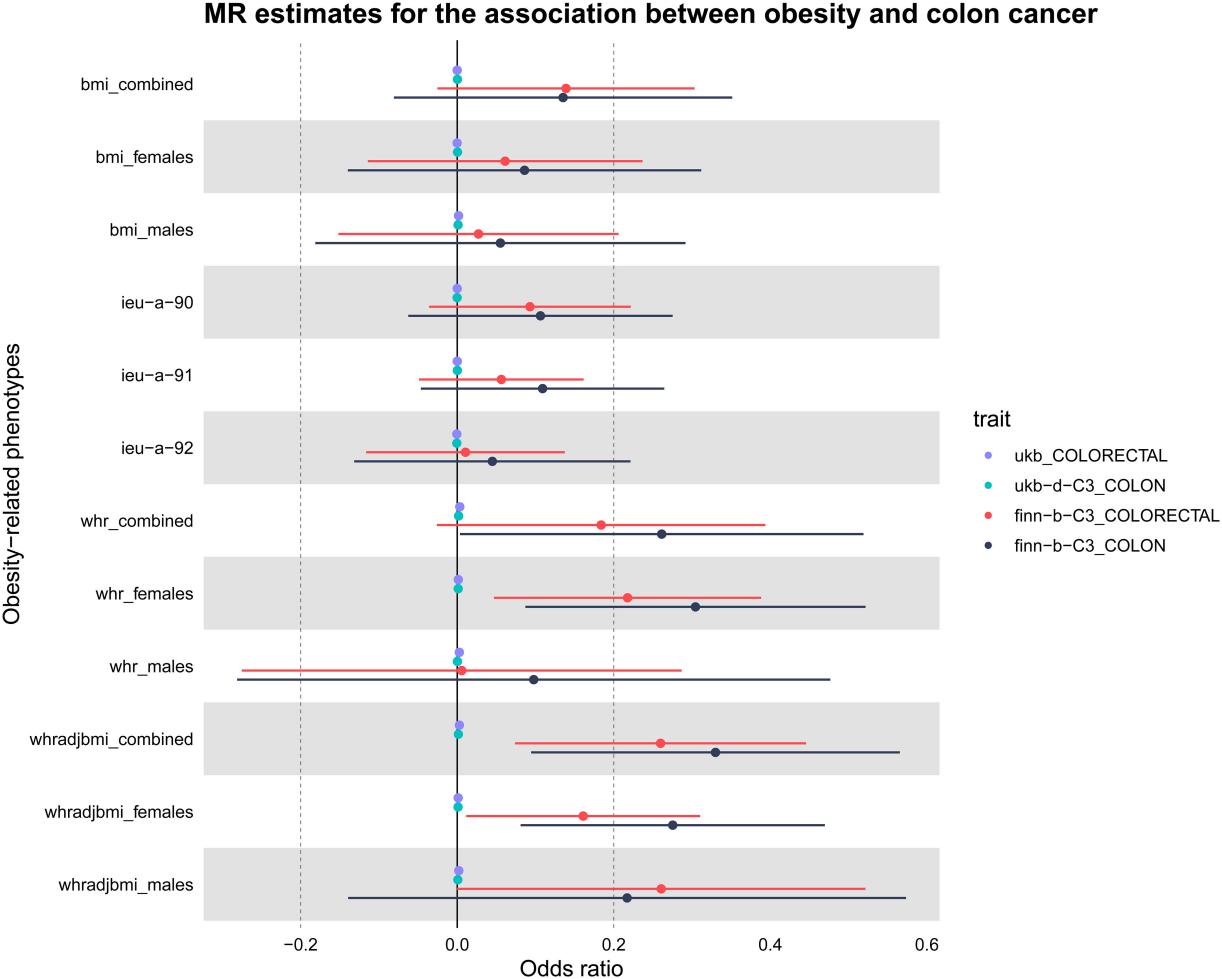
Presentation of MR estimations in a Forest plot concerning obesity-related determinants and colorectal cancer. The forest plot depicts both sex-aggregated and sex-stratified IVW estimates. Data were sourced from four independent cohorts. Notably, significant associations with colorectal cancer risk were identified exclusively for WHR, particularly in the female subgroup.

**Figure 3:**
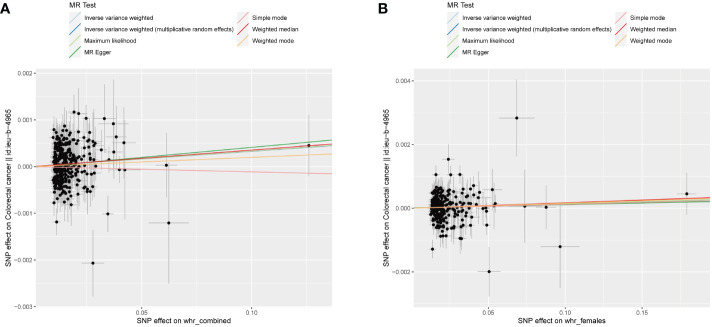
WHR scatter diagrams concerning colorectal malignancy susceptibility. **(A)** Combined-sex WHR scatter plot reveals a direct positive correlation with the incidence of colorectal malignancy. **(B)** Female-specific WHR scatter plot revealing a significant association with colorectal cancer. Both plots were generated using an IVW random-effects model and showed no significant pleiotropic effects (intercept_pval > 0.05). Potential dataset heterogeneity (Q_pval < 0.05) did not significantly alter the results.

The study’s schematic representation (**Figure 1**) and its elaboration within the Methods segment indicate that chosen SNPs tied to obesity phenotypes were employed as exposure determinants. In tandem, four colorectal cancer-associated cohorts served as the outcome determinants for Mendelian randomization. Relevant P-values for these models were determined. From the outcomes procured via IVW strategies, no marked causal linkage was discerned between BMI and colorectal cancer. Yet, a notable link was identified between WHR and its adjusted variant (WHRadjBMI) and colorectal cancer, implying WHR’s potential as a colorectal cancer risk determinant. Remarkably, gender-specific findings revealed this significant association only in the female segment, absent in males, suggesting an amplified colorectal cancer risk for females exhibiting a raised WHR (**Figure 2** and **Table 2**, noteworthy outcomes emphasized in red). Scatter diagrams further attested to the risk that heightened WHR imposes concerning colorectal cancer’s initiation and advancement (**Figure 3**). Additional Mendelian randomization analyses for heterogeneity and pleiotropy were also performed. It was observed that no pleiotropic effects were present in the data (intercept_pval > 0.05). While potential heterogeneity could be noted in some data sets for WHR and WHRadjBMI in relation to colorectal cancer (Q_pval < 0.05), such heterogeneity did not significantly impact the results, as evidenced by the IVW random-effects model.

**Table 2:** Mendelian randomization analysis results for obesity-related risk factors in colorectal cancer across different cohorts.

id.exposure	id.outcome	method	nsnp	b	OR (95% CI)	P value
ieu-a-90	finn-b-C3_COLON	Inverse variance weighted	17	0.106	1.11 (0.94 to 1.32)	2.17E-01
	finn-b-C3_COLORECTAL		17	0.093	1.1 (0.96 to 1.25)	1.57E-01
	ieu-b-4965		16	0.000	1 (1 to 1)	9.87E-01
	ukb-d-C3_COLON		17	0.000	1 (1 to 1)	8.25E-01
ieu-a-91	finn-b-C3_COLON	Inverse variance weighted	11	0.109	1.12 (0.95 to 1.3)	1.69E-01
	finn-b-C3_COLORECTAL		11	0.056	1.06 (0.95 to 1.18)	2.93E-01
	ieu-b-4965		11	0.000	1 (1 to 1)	9.59E-01
	ukb-d-C3_COLON		11	0.000	1 (1 to 1)	7.24E-01
ieu-a-92	finn-b-C3_COLON	Inverse variance weighted	2	0.045	1.05 (0.88 to 1.25)	6.18E-01
	finn-b-C3_COLORECTAL		2	0.011	1.01 (0.89 to 1.15)	8.70E-01
	ieu-b-4965		2	-0.001	1 (1 to 1)	4.42E-01
	ukb-d-C3_COLON		2	-0.001	1 (1 to 1)	2.51E-01
bmi_combined	finn-b-C3_COLON	Inverse variance weighted	525	0.135	1.14 (0.92 to 1.42)	2.20E-01
	finn-b-C3_COLORECTAL		525	0.139	1.15 (0.98 to 1.35)	9.72E-02
	ieu-b-4965		525	0.000	1 (1 to 1)	9.47E-01
	ukb-d-C3_COLON		530	0.000	1 (1 to 1)	6.24E-01
bmi_females	finn-b-C3_COLON	Inverse variance weighted	308	0.086	1.09 (0.87 to 1.37)	4.55E-01
	finn-b-C3_COLORECTAL		308	0.061	1.06 (0.89 to 1.27)	4.94E-01
	ieu-b-4965		309	0.000	1 (1 to 1)	8.64E-01
	ukb-d-C3_COLON		313	0.000	1 (1 to 1)	5.38E-01
bmi_males	finn-b-C3_COLON	Inverse variance weighted	255	0.055	1.06 (0.83 to 1.34)	6.47E-01
	finn-b-C3_COLORECTAL		255	0.027	1.03 (0.86 to 1.23)	7.66E-01
	ieu-b-4965		259	0.002	1 (1 to 1)	8.80E-02
	ukb-d-C3_COLON		263	0.001	1 (1 to 1)	8.12E-02
whr_combined	finn-b-C3_COLON	Inverse variance weighted	337	0.261	1.3 (1 to 1.68)	4.70E-02
	finn-b-C3_COLORECTAL		337	0.184	1.2 (0.97 to 1.48)	8.58E-02
	ieu-b-4965		336	0.003	1 (1 to 1.01)	6.34E-03
	ukb-d-C3_COLON		345	0.002	1 (1 to 1)	2.80E-02
whr_females	finn-b-C3_COLON	Inverse variance weighted	242	0.304	1.36 (1.09 to 1.68)	6.04E-03
	finn-b-C3_COLORECTAL		242	0.218	1.24 (1.05 to 1.47)	1.24E-02
	ieu-b-4965		240	0.001	1 (1 to 1)	1.86E-01
	ukb-d-C3_COLON		245	0.001	1 (1 to 1)	4.94E-02
whr_males	finn-b-C3_COLON	Inverse variance weighted	104	0.098	1.1 (0.75 to 1.61)	6.13E-01
	finn-b-C3_COLORECTAL		104	0.006	1.01 (0.76 to 1.33)	9.68E-01
	ieu-b-4965		103	0.003	1 (1 to 1.01)	1.00E-01
	ukb-d-C3_COLON		106	0.000	1 (1 to 1)	7.87E-01
whradjbmi_combined	finn-b-C3_COLON	Inverse variance weighted	314	0.330	1.39 (1.1 to 1.76)	6.03E-03
	finn-b-C3_COLORECTAL		314	0.260	1.3 (1.08 to 1.56)	6.16E-03
	ieu-b-4965		313	0.003	1 (1 to 1)	8.48E-03
	ukb-d-C3_COLON		318	0.002	1 (1 to 1)	3.05E-02
whradjbmi_females	finn-b-C3_COLON	Inverse variance weighted	269	0.275	1.32 (1.08 to 1.6)	5.48E-03
	finn-b-C3_COLORECTAL		269	0.161	1.17 (1.01 to 1.36)	3.47E-02
	ieu-b-4965		265	0.001	1 (1 to 1)	1.51E-01
	ukb-d-C3_COLON		271	0.001	1 (1 to 1)	5.06E-02
whradjbmi_males	finn-b-C3_COLON	Inverse variance weighted	120	0.217	1.24 (0.87 to 1.77)	2.33E-01
	finn-b-C3_COLORECTAL		120	0.261	1.3 (1 to 1.68)	5.02E-02
	ieu-b-4965		121	0.002	1 (1 to 1)	1.80E-01
	ukb-d-C3_COLON		121	0.001	1 (1 to 1)	4.21E-01

### Reverse Mendelian randomization

**Figure 4:**
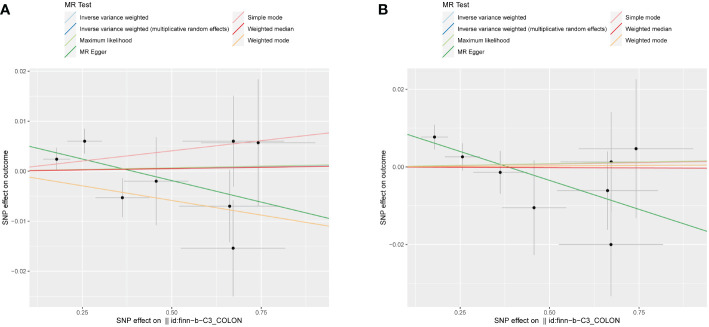
Scatter plots illustrating the absence of a reverse causal relationship between colorectal cancer and obesity-related phenotypes. **(A)** Combined-sex scatter plot of BMI showing no significant relationship with colorectal cancer. **(B)** Female-specific scatter plot of WHR also revealing no significant association with colorectal cancer. Both plots were generated using an IVW method and showed no statistical significance, thereby refuting a reverse causal link between colorectal cancer and obesity-related phenotypes.

In order to evaluate the causal relationship linking obesity-linked phenotypes and colorectal cancer, critical genetic loci from genome-wide association research focused on colorectal cancer served as exposure benchmarks. On the flip side, phenotypes associated with obesity acted as the outcome variables in a dual-sample Mendelian analysis. Considering there were no significant loci at a P-value cutoff of 5e-8, this cutoff got shifted to 5e-6, keeping the rest of the conditions unchanged (33). Through the application of the IVW approach, it was determined that there was no statistical significance in all reverse Mendelian randomization evaluations. Consequently, a reverse causal relationship was negated, as depicted in **Figure 4**. Consequently, the onset of colorectal cancer does not contribute to obesity or related phenotypes (**Table 3**).

**Table 3:** Summary of reverse Mendelian randomization analyses investigating the potential causal link between colorectal cancer and obesity-related phenotypes.

id.exposure	id.outcome	method	nsnp	b	se	pval
finn-b-C3_COLON	bmi_combined	Inverse variance weighted	8	0.001	0.006	8.36E-01
finn-b-C3_COLON	bmi_females	Inverse variance weighted	8	-0.004	0.007	5.92E-01
finn-b-C3_COLON	bmi_males	Inverse variance weighted	8	0.007	0.007	3.03E-01
finn-b-C3_COLON	whr_combined	Inverse variance weighted	8	0.004	0.007	5.94E-01
finn-b-C3_COLON	whr_females	Inverse variance weighted	8	0.002	0.007	8.31E-01
finn-b-C3_COLON	whr_males	Inverse variance weighted	8	0.006	0.008	4.37E-01
finn-b-C3_COLON	whradjbmi_combined	Inverse variance weighted	8	0.004	0.006	5.26E-01
finn-b-C3_COLON	whradjbmi_females	Inverse variance weighted	8	0.004	0.007	5.46E-01
finn-b-C3_COLON	whradjbmi_males	Inverse variance weighted	8	0.001	0.007	8.29E-01
finn-b-C3_COLORECTAL	bmi_combined	Inverse variance weighted	12	0.002	0.005	7.57E-01
finn-b-C3_COLORECTAL	bmi_females	Inverse variance weighted	12	-0.002	0.005	6.21E-01
finn-b-C3_COLORECTAL	bmi_males	Inverse variance weighted	12	0.006	0.008	4.46E-01
finn-b-C3_COLORECTAL	ieu-a-90	Inverse variance weighted	5	0.051	0.045	2.57E-01
finn-b-C3_COLORECTAL	ieu-a-91	Inverse variance weighted	5	0.064	0.069	3.49E-01
finn-b-C3_COLORECTAL	ieu-a-92	Inverse variance weighted	5	0.055	0.208	7.92E-01
finn-b-C3_COLORECTAL	whr_combined	Inverse variance weighted	12	-0.001	0.007	8.79E-01
finn-b-C3_COLORECTAL	whr_females	Inverse variance weighted	12	-0.005	0.008	5.32E-01
finn-b-C3_COLORECTAL	whr_males	Inverse variance weighted	12	0.002	0.010	8.28E-01
finn-b-C3_COLORECTAL	whradjbmi_combined	Inverse variance weighted	12	-0.002	0.007	7.33E-01
finn-b-C3_COLORECTAL	whradjbmi_females	Inverse variance weighted	12	-0.004	0.009	6.55E-01
finn-b-C3_COLORECTAL	whradjbmi_males	Inverse variance weighted	12	-0.001	0.007	9.17E-01
ieu-b-4965	bmi_combined	Inverse variance weighted	28	-0.286	0.239	2.31E-01
ieu-b-4965	bmi_females	Inverse variance weighted	28	-0.397	0.303	1.90E-01
ieu-b-4965	bmi_males	Inverse variance weighted	28	-0.242	0.389	5.35E-01
ieu-b-4965	ieu-a-90	Inverse variance weighted	17	-2.946	1.905	1.22E-01
ieu-b-4965	ieu-a-91	Inverse variance weighted	17	-1.558	2.969	6.00E-01
ieu-b-4965	ieu-a-92	Inverse variance weighted	17	-4.601	5.435	3.97E-01
ieu-b-4965	whr_combined	Inverse variance weighted	28	0.194	0.302	5.20E-01
ieu-b-4965	whr_females	Inverse variance weighted	28	-0.039	0.386	9.20E-01
ieu-b-4965	whr_males	Inverse variance weighted	28	0.492	0.407	2.27E-01
ieu-b-4965	whradjbmi_combined	Inverse variance weighted	28	0.370	0.361	3.05E-01
ieu-b-4965	whradjbmi_females	Inverse variance weighted	28	0.122	0.464	7.94E-01
ieu-b-4965	whradjbmi_males	Inverse variance weighted	28	0.812	0.471	8.47E-02
ukb-d-C3_COLON	bmi_combined	Inverse variance weighted	22	-0.230	0.661	7.28E-01
ukb-d-C3_COLON	bmi_females	Inverse variance weighted	22	-0.526	0.832	5.27E-01
ukb-d-C3_COLON	bmi_males	Inverse variance weighted	22	-0.231	0.697	7.40E-01
ukb-d-C3_COLON	ieu-a-90	Inverse variance weighted	9	-2.415	6.263	7.00E-01
ukb-d-C3_COLON	ieu-a-91	Inverse variance weighted	9	0.042	9.347	9.96E-01
ukb-d-C3_COLON	ieu-a-92	Inverse variance weighted	7	5.909	14.786	6.89E-01
ukb-d-C3_COLON	whr_combined	Inverse variance weighted	22	-0.075	0.585	8.98E-01
ukb-d-C3_COLON	whr_females	Inverse variance weighted	22	0.123	0.693	8.60E-01
ukb-d-C3_COLON	whr_males	Inverse variance weighted	22	-0.318	0.737	6.66E-01
ukb-d-C3_COLON	whradjbmi_combined	Inverse variance weighted	22	0.118	0.790	8.82E-01
ukb-d-C3_COLON	whradjbmi_females	Inverse variance weighted	22	0.406	0.841	6.30E-01
ukb-d-C3_COLON	whradjbmi_males	Inverse variance weighted	22	-0.123	1.054	9.07E-01

### Genetic correlation between colorectal cancer and obesity-related phenotypes

**Figure 5:**
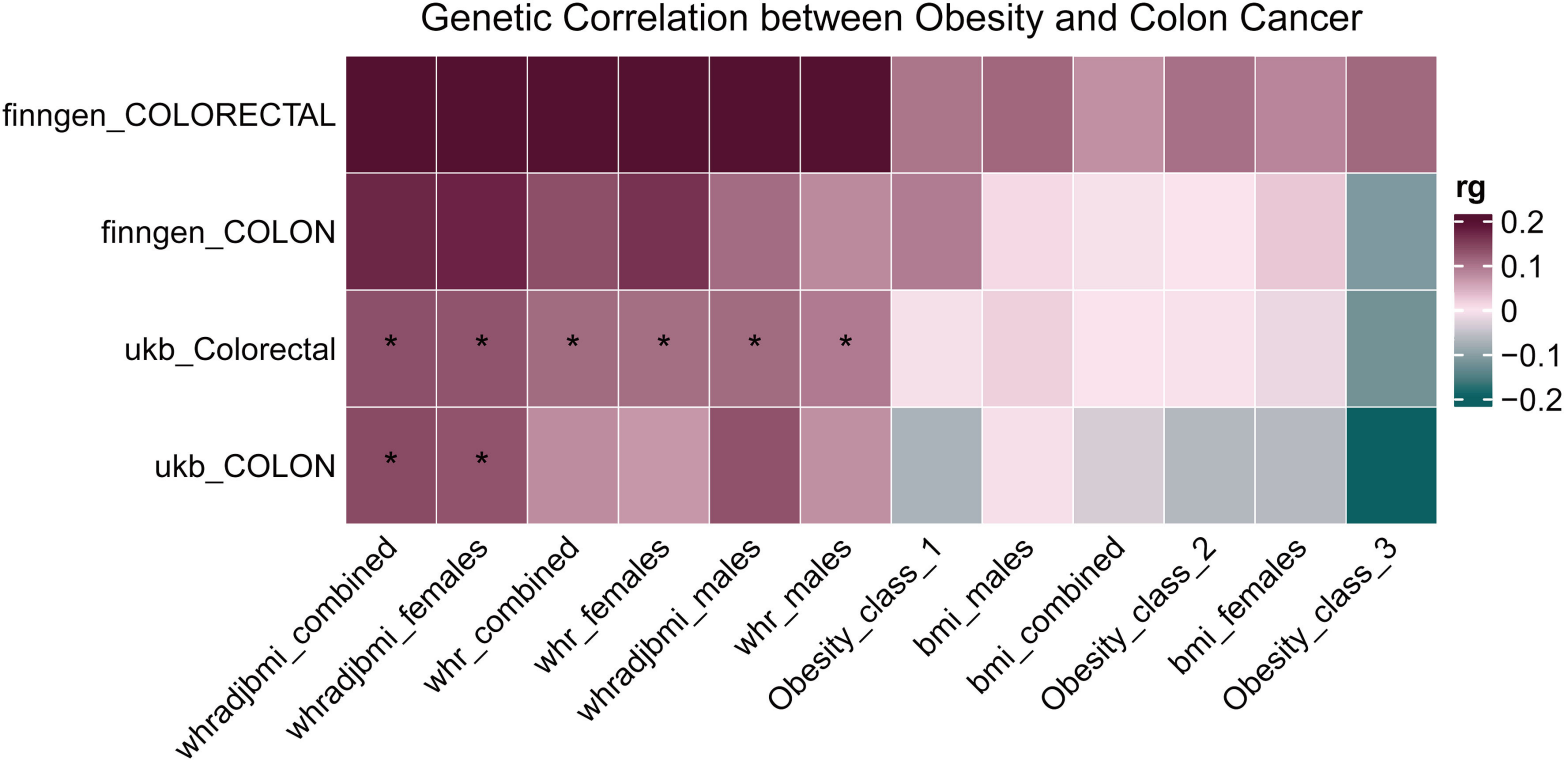
Heatmap of genetic correlation between colorectal cancer and obesity-related phenotypes. The heatmap represents the genetic correlation assessed through LDSC between colorectal cancer data sourced from the UK Biobank and various obesity-related phenotypes. Statistically significant correlations are denoted with an asterisk. *P < 0.05.

Using the provided methodologies, a study was carried out to explore the genetic connection of colorectal cancer to obesity-related phenotypes. A genetic correlation analysis (rg) was conducted on the aforementioned datasets using LDSC. The results indicated a notable genetic correlation between colorectal cancer data from the UK Biobank and WHR measurements, achieving statistical significance (*P* < 0.05, denoted with an asterisk). Furthermore, an elevated genetic correlation (rg) was observed overall between WHR and colorectal cancer, as illustrated in **Figure 5**. Upon examination, a genetic correlation of 0.13 emerged between colorectal cancer and WHR, factoring in BMI adjustments (WHRadjBMI); the significance of this correlation is statistically supported (*P* = 1.500E-03) in **Table 4**.

**Table 4:** Genetic correlation between colorectal cancer and obesity-related phenotypes.

p1	p2	rg	se	z	p
Obesity_class_1	finngen_COLON	0.09	0.13	0.73	4.672E-01
	finngen_COLORECTAL	0.10	0.16	0.61	5.437E-01
	ukb_COLON	-0.07	0.07	-1.01	3.103E-01
	ukb_Colorectal	-0.01	0.06	-0.12	9.011E-01
Obesity_class_2	finngen_COLON	0.00	0.15	0.00	9.978E-01
	finngen_COLORECTAL	0.10	0.19	0.54	5.926E-01
	ukb_COLON	-0.07	0.09	-0.73	4.624E-01
	ukb_Colorectal	0.00	0.08	-0.03	9.764E-01
Obesity_class_3	finngen_COLON	-0.11	0.24	-0.47	6.367E-01
	finngen_COLORECTAL	0.11	0.24	0.46	6.478E-01
	ukb_COLON	-0.23	0.14	-1.60	1.091E-01
	ukb_Colorectal	-0.12	0.12	-1.01	3.104E-01
whr_combined	finngen_COLON	0.14	0.09	1.55	1.202E-01
	finngen_COLORECTAL	0.29	0.17	1.71	8.740E-02
	ukb_COLON	0.08	0.05	1.58	1.137E-01
	ukb_Colorectal	0.11	0.04	2.81	4.900E-03
whr_females	finngen_COLON	0.16	0.09	1.73	8.280E-02
	finngen_COLORECTAL	0.26	0.16	1.64	1.007E-01
	ukb_COLON	0.07	0.05	1.28	2.006E-01
	ukb_Colorectal	0.11	0.04	2.52	1.190E-02
whr_males	finngen_COLON	0.08	0.09	0.93	3.547E-01
	finngen_COLORECTAL	0.28	0.18	1.61	1.072E-01
	ukb_COLON	0.08	0.06	1.35	1.755E-01
	ukb_Colorectal	0.09	0.04	2.10	3.580E-02
whradjbmi_combined	finngen_COLON	0.17	0.10	1.80	7.210E-02
	finngen_COLORECTAL	0.31	0.18	1.70	8.980E-02
	ukb_COLON	0.14	0.06	2.27	2.320E-02
	ukb_Colorectal	0.13	0.04	3.17	1.500E-03
whradjbmi_females	finngen_COLON	0.18	0.10	1.83	6.740E-02
	finngen_COLORECTAL	0.27	0.16	1.63	1.026E-01
	ukb_COLON	0.13	0.06	2.09	3.700E-02
	ukb_Colorectal	0.13	0.04	2.91	3.600E-03
whradjbmi_males	finngen_COLON	0.11	0.10	1.07	2.855E-01
	finngen_COLORECTAL	0.27	0.18	1.51	1.311E-01
	ukb_COLON	0.13	0.07	1.90	5.710E-02
	ukb_Colorectal	0.11	0.05	2.08	3.710E-02
bmi_combined	finngen_COLON	0.00	0.07	-0.07	9.440E-01
	finngen_COLORECTAL	0.07	0.09	0.83	4.045E-01
	ukb_COLON	-0.04	0.05	-0.76	4.465E-01
	ukb_Colorectal	0.00	0.04	0.00	9.975E-01
bmi_females	finngen_COLON	0.03	0.08	0.33	7.436E-01
	finngen_COLORECTAL	0.08	0.10	0.84	4.004E-01
	ukb_COLON	-0.06	0.06	-1.12	2.644E-01
	ukb_Colorectal	-0.02	0.04	-0.41	6.829E-01
bmi_males	finngen_COLON	0.01	0.08	0.14	8.913E-01
	finngen_COLORECTAL	0.11	0.11	1.05	2.929E-01
	ukb_COLON	-0.01	0.05	-0.12	9.034E-01
	ukb_Colorectal	0.02	0.04	0.42	6.719E-01

Statistically significant P-values are highlighted in red.

### Mendelian analysis concerning risk determinants for colorectal cancer

**Figure 6:**
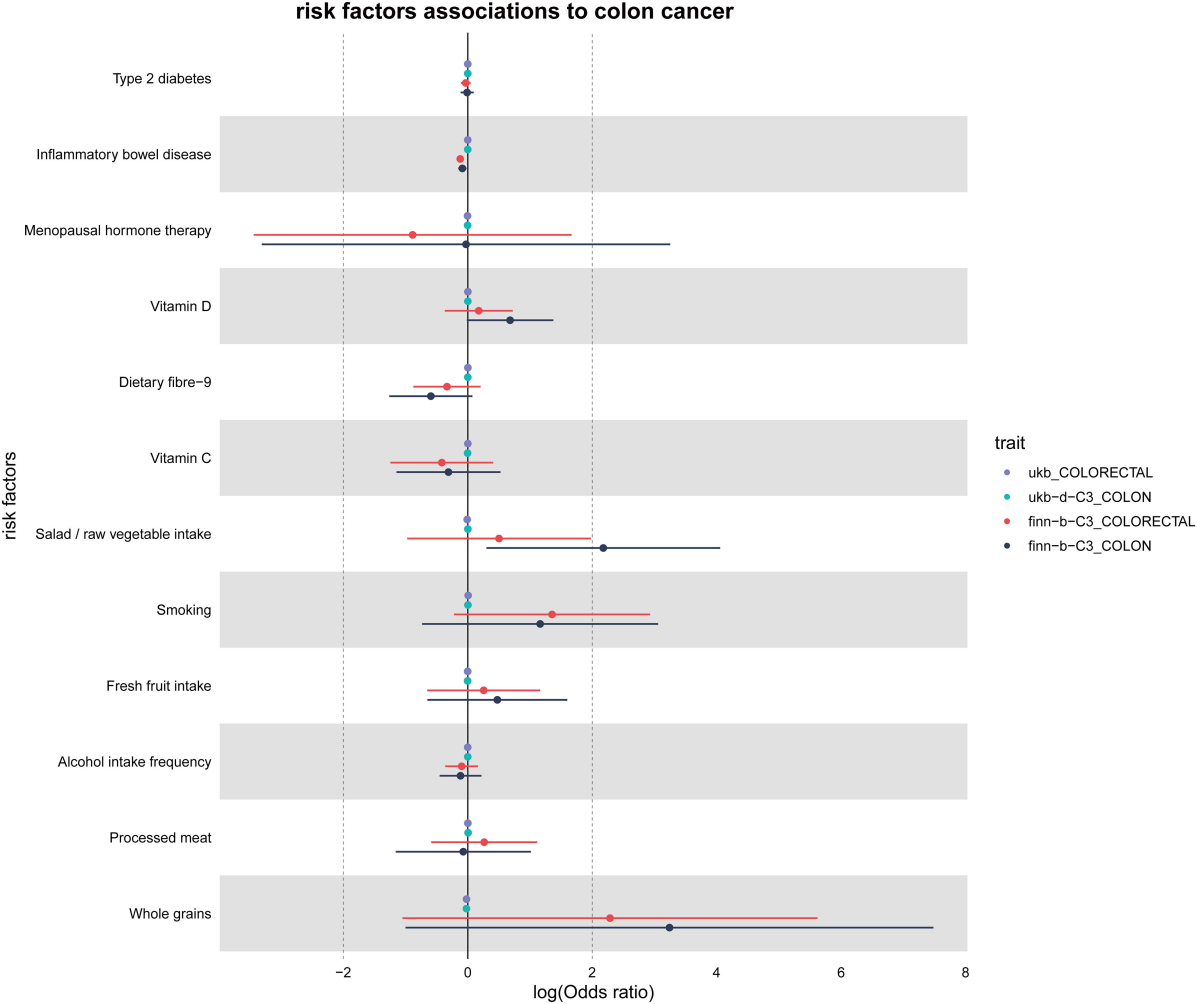
MR results for risk factors associated with colorectal cancer. MR results identify inflammatory bowel disease as a protective factor and salad/raw vegetable intake as a risk factor.

In alignment with the delineated procedure, a study was undertaken to elucidate the correlation between certain risk determinants and colorectal cancer prevalence. These determinants were utilized as exposure indices, with colorectal cancer identified as the outcome index. Based on the quantity of instrumental variables, suitable linear models were determined, producing the MR outcomes illustrated in **Figure 6**. Upon setting a *P*-value threshold at < 0.05, inflammatory bowel disease was identified as a potential protective mechanism against colorectal cancer. Conversely, consumption of salads and raw vegetables is identified as a potential hazard. Statistical significance was not found for the other risk determinants analyzed.

## Discussion

The global ascent of obesity and its prospective consequences for various malignancies, including CRC, has emerged as a focal point of research. Over recent decades, there has been a marked escalation in the BMI of adults worldwide (4). The WHO statistics has substantiated this upward trajectory, indicating that close to 1.5 billion adults globally were overweight, of which a concerning 500 million were categorized as obese (5). Notably, a parallel trend has been observed between the rise in obesity rates and the increased incidence of CRC (2, 6). Numerous studies have further substantiated a definitive relationship between obesity and CRC risk (8, 34, 35). Consistent findings indicating a decreased risk of CRC following weight loss surgeries have been delineated in studies conducted in Italy (36), England (37), and the United States (38). Furthermore, of clinical significance, the research direction has shifted recently, with studies beginning to examine the prognostic value of obesity on post-diagnosis survival rates for CRC patients (39, 40). Through genetic predictive investigations, Papadimitriou et al. discerned a relationship between weight during early childhood (around 10 years of age) and elevated risks of CRC, notably with a significant rise in distal colon cancer risks (41). In obese East Asian populations, studies spearheaded by Kwon et al. highlighted that overweight status and obesity emerge as prominent determinants in the progression of colorectal tumors (42). Similar to findings in European populations, Suzuki et al. identified in Asians a discernible positive correlation between elevated BMI and CRC risk (43, 44). Current dominant research findings, despite discrepancies, have consistently associated elevated BMI with poorer survival outcomes (16, 45, 46).

BMI, traditionally viewed as a cornerstone in the evaluation of obesity-cancer correlations, failed to display a causative link with colorectal cancer in our MR analysis. This result aligned with previous studies, notably by Yamamoto-Honda et al., that found no significant association between BMI and CRC, especially within Asian groups (47). Moreover, research conducted by Zhai et al. suggested that in colorectal cancer patients, it is visceral obesity, not BMI, that provided a more precise evaluation of obesity’s influence on metabolic and postoperative infectious complications (48). To delve into other obesity-related risk indicators, our two-sample MR approach sourced data from the IEU Open GWAS Project, Zenodo, and FinnGen. Interestingly, central obesity metrics, specifically WHR and its adjusted variant (WHRadjBMI), demonstrated pronounced causal relationships with CRC, suggesting their potential significance in evaluating risk. In line with this perspective, Hashemi Madani et al. found that there was a notable link between WHR and elevated risks of cancers in the colon and stomach among females. This highlights the pivotal importance of central obesity, particularly the distribution of abdominal fat, in influencing certain cancer susceptibilities (49). Similarly, studies by Mili et al. and Safizadeh et al. substantiated those measures of central obesity, including waist circumference and WHR, provided more robust and consistent predictions of CRC incidence compared to BMI (50, 51). The link between central obesity and CRC incidence highlights the importance of considering gender differences. Prior investigations underscored notable gender discrepancies within the genetic mechanisms governing body metrics, particularly BMI and WHR, likely modulated by reproductive hormones (52, 53). Notably, research by Mureșan et al. indicated that women exhibit increased oxidative stress markers and reduced homocysteine levels during the later stages of pregnancy, suggesting that these gender-specific metabolic changes during critical periods could influence long-term cancer risks (54). Distinctive findings of this study, utilizing the IVW method, demonstrated a marked relationship between increased WHR and susceptibility to colorectal cancer, notably in females, without any evident connection in males. Observational studies consistently revealed an association between augmented BMI and CRC risk, with gender serving as a potential modulator (9). With a spotlight on gender discrepancies, Ortega et al. found CRC risk tethered to both overall and abdominal fat in men, whereas in women, the risk was solely attributed to WHR (55). Consistently, employing Mendelian randomization, Bull et al. established a direct correlation of heightened BMI with male colon cancer risk, contrasting with a stronger WHR association in females (56). Hashemi Madani et al. also observed that WHR significantly correlated with colon and gastric cancer risks in females, unlike in males, suggesting a gender-specific interplay between obesity metrics and cancer risks (49). Interestingly, Thrift et al., via Mendelian randomization, pinpointed an elevated BMI as a potent risk for colon cancer in females, but such an association was elusive in males when BMI was genetically determined (44). Similarly, Loh et al. brought attention to the WHRadjBMI, discerning a notable relationship between an increase in WHRadjBMI and augmented colorectal cancer potential in females; such a connection proved elusive for males (57).

The observed gender-specific associations necessitated further exploration of the underlying biological mechanisms. Elevated abdominal adiposity, displaying strong associations with elements including hyperinsulinemia, anomalies in insulin function, and modifications in systems of the IGF realm, might have influenced the probability of colorectal cancer development and the resulting fatality rates (58, 59). Interestingly, according to Trevisan et al., there exists a distinct gender-based variation, with males showing a more pronounced association, when connecting elevated body mass to colorectal cancer deaths, influenced by conditions such as hyperinsulinemia and insulin resistance (60). The discerned correlation aligned with the prevailing scientific understanding that links obesity-induced colorectal cancer susceptibility to processes such as disruptions in glucose metabolism, insulin-like growth factors, gender-related hormones, peptides derived from adipose tissue, markers of inflammatory responses, and oxidative stress (61, 62). Interestingly, a Swedish clinical research highlighted a connection between leptin metrics and CRC likelihood in males; however, such a connection wasn’t evident among females (63). Additionally, certain leptin gene SNPs increased CRC susceptibility in females regardless of obesity, whereas a specific ADIPOQ SNP manifested only in obese males, suggesting gender-specific CRC predispositions (64). Furthermore, the occurrence of such gender-based variations might have been associated with sex hormone fluctuations. According to Slattery et al., estrogen exposure was protective against MSI+ colon tumors in women; however, its insufficiency in the elderly female population increased tumor susceptibility (65). Concurrently, Bell et al. reported that elevated triglyceride levels correlated with increased BMI in males but with heightened WHR in females, highlighting gender-specific metabolic implications influenced by adiposity patterns and hormonal profiles (66). Moreover, elevated serum markers such as CRP-1, TNF-α, and IL-6, indicative of persistent inflammation, have been linked to CRC progression, with this association appearing stronger in males (67, 68). Overall, gender differences appeared to influence the link between obesity indicators and a predisposition to CRC, underscoring the need for targeted research.

The research brought to light correlations between obesity-driven phenotypes and the predisposition to colorectal cancer, most notably emphasizing the relationships concerning WHR, WHRadjBMI, and female incidences. These findings emphasized the need for strategic screening among individuals with elevated WHR and underscored the value of diversified datasets in subsequent research endeavors. However, certain constraints in this investigation warranted attention. Primary data were sourced from the IEU Open GWAS Project and Zenodo. Despite their comprehensiveness, it remains plausible that these repositories do not fully capture global genetic variations, thus posing challenges to the universal applicability of the results. Efforts were made to control for recognized confounding factors; however, the possibility of unaccounted variables remains. The validity of the instrumental variables selected depended on the strength and consistency of the primary GWAS studies, which introduces the possibility of biases influencing the MR findings. Significant heterogeneity was identified in the datasets related to WHR and WHRadjBMI, introducing additional complexity without substantially altering the primary findings. Owing to the inherent observational character of MR analyses, the research could be vulnerable to certain limitations, including pleiotropy. Possibilities of dataset intersection or undisclosed associations, particularly in the context of the UK Biobank and FinnGen samples, could have existed. Moreover, while extensive GWAS datasets were considered, the unavailability of key datasets could have impeded the detailed evaluation of colorectal cancer attributes. Nevertheless, the study demonstrated a significant association between WHR, WHRadjBMI, and the incidence of colorectal cancer, predominantly in females. Based on these findings, there is a pronounced need for enhanced screening procedures for individuals with elevated WHR values. Moreover, the results underscore the imperative of incorporating broader and more diverse datasets in subsequent research.

## Conclusion

In a bidirectional MR examination focused on obesity-related phenotypes pertaining to colorectal cancer, complemented by genetic correlation data, a causal connection between obesity’s influence and colorectal cancer was delineated. There wasn’t any pronounced association involving grade 3 obesity parameters and BMI when considering colorectal cancer. Nonetheless, a notable causal relationship existed between WHR and WHRadjBMI, emphasizing a distinct gender variation, with a higher prevalence in females. Furthermore, a potent genetic correlation between both WHR metrics and colorectal cancer was corroborated, reinforcing the causal inference. Collectively, these findings suggest that an increased WHR acts as a significant predictor, enhancing the risk for colorectal cancer development and progression. Clinically, attention is focused on the critical importance WHR measurements play in assessing CRC vulnerability, notably significant among females. Integration of WHR evaluations into standard clinical practice is recommended, enabling prompt interventions and providing guidance on behavioral modifications. In summary, recognizing the significance of increased WHR offers a vital perspective for both clinical considerations and early interventions in colorectal cancer.

## Data availability statement

The datasets presented in this study can be found in online repositories. The names of the repository/repositories and accession number(s) can be found below: https://gwas.mrcieu.ac.uk/, ieu-a-90, ieu-a-91, ieu-a-92, ieu-b-4965, ukb-d-C3_COLON, ukb-b-223, ukb-b-6324, ukb-b-5779, ukb-b-1996, ukb-b-3881, ukb-d-1448_3, ukb-b-19085, ukb-b-19390, Dukb-b-18593, ukb-b-7043, ukb-b-18541, ebi-a-GCST006867, ieu-a-294, https://www.finngen.fi/en, finn-b-C3_COLON, finn-b-C3_COLORECTAL.

## Ethics statement

Ethical approval was not required for the study involving humans in accordance with the local legislation and institutional requirements. Written informed consent to participate in this study was not required from the participants or the participants’ legal guardians/next of kin in accordance with the national legislation and the institutional requirements.

## Author contributions

XC: Formal analysis, Methodology, Writing – original draft, Writing – review & editing. MY: Data curation, Supervision, Writing – review & editing. WZ: Investigation, Resources, Writing – review & editing. JT: Investigation, Resources, Writing – review & editing. QL: Data curation, Resources, Supervision, Writing – review & editing. XY: Conceptualization, Funding acquisition, Supervision, Writing – review & editing.
